# A kinetic model of the evolution of a protein interaction network

**DOI:** 10.1186/1471-2164-14-172

**Published:** 2013-03-14

**Authors:** Piotr H Pawlowski, Szymon Kaczanowski, Piotr Zielenkiewicz

**Affiliations:** 1 Institute of Biochemistry and Biophysics of the Polish Academy of Sciences, Pawińskiego 5a, 02-106, Warszawa, Poland; 2 Laboratory of Plant Molecular Biology, Warsaw University, Pawińskiego 5a, 02-106, Warszawa, Poland

## Abstract

**Background:**

Known protein interaction networks have very particular properties. Old proteins tend to have more interactions than new ones. One of the best statistical representatives of this property is the node degree distribution (distribution of proteins having a given number of interactions). It has previously been shown that this distribution is very close to the sum of two distinct exponential components. In this paper, we asked: What are the possible mechanisms of evolution for such types of networks? To answer this question, we tested a kinetic model for simplified evolution of a protein interactome. Our proposed model considers the emergence of new genes and interactions and the loss of old ones. We assumed that there are generally two coexisting classes of proteins. Proteins constituting the first class are essential only for ecological adaptations and are easily lost when ecological conditions change. Proteins of the second class are essential for basic life processes and, hence, are always effectively protected against deletion. All proteins can transit between the above classes in both directions. We also assumed that the phenomenon of gene duplication is always related to ecological adaptation and that a new copy of a duplicated gene is not essential. According to this model, all proteins gain new interactions with a rate that preferentially increases with the number of interactions (the rich get richer). Proteins can also gain interactions because of duplication. Proteins lose their interactions both with and without the loss of partner genes.

**Results:**

The proposed model reproduces the main properties of protein-protein interaction networks very well. The connectivity of the oldest part of the interaction network is densest, and the node degree distribution follows the sum of two shifted power-law functions, which is a theoretical generalization of the previous finding. The above distribution covers the wide range of values of node degrees very well, much better than a power law or generalized power law supplemented with an exponential cut-off. The presented model also relates the total number of interactome links to the total number of interacting proteins. The theoretical results were for the interactomes of *A. thaliana, B. taurus, C. elegans, D. melanogaster, E. coli, H. pylori, H. sapiens, M. musculus, R. norvegicus* and *S. cerevisiae*.

**Conclusions:**

Using these approaches, the kinetic parameters could be estimated. Finally, the model revealed the evolutionary kinetics of proteome formation, the phenomenon of protein differentiation and the process of gaining new interactions.

## Background

Although an evolutionary viewpoint in network studies is not a new concept [1], it still gains new followers [2], especially in the field of the evolution of protein interactions [3,4] and in regulatory [5] and metabolic [6] networks. Investigators of protein–protein interaction (PPI) networks indicate that functional evolution [7], modular organization [8], evolutionary pressures [9] and genome duplications [10,11] are crucial factors in shaping network architecture, and several of these researchers negatively correlate the connectivity of well-conserved proteins in the network with their individual rate of evolution [12,13]. Numerous studies indicate that local network growth rules, such as gene duplication and gene diversification, can give rise to scale-free connectivity distributions and an effective linear preferential attachment [14]. Original approaches, such as the evolutionary excess retention method [15] or modeling of protein evolution using a lattice representation of their structures, were proposed to determine the effect of explicit selection on PPI [16]. The most popular ideas for the main mechanisms for generating the scale-free, older core and hierarchically modular topology of protein interaction networks are the Barabasi-Albert “the rich get richer” model of preferential attachment and the gene duplication and divergence model of Ispolatov et al. [17]. They were recently criticized on the basis of the Kim-Marcotte stochastic crystal growth model [18], which captures the age-dependency of the interaction density along with the hierarchical modularity. Nevertheless, some of the elements of the previous models can still be beneficial in modeling the overall kinetics of interactome evolution.

Consequently, one may expect that the current network architecture may provide quantitative information about the network history. Comparing the presented kinetic model for the evolution of the protein interaction network with the data for *S. cerevisiae* and nine other species allows us to estimate the rates of the basic processes of interactome evolution, i.e., the emergence of new genes and the loss of the old ones, the duplication phenomenon, the differentiation of functional significance, the obtaining of new interactions and the deactivation of active ones.

**Figure 1:**
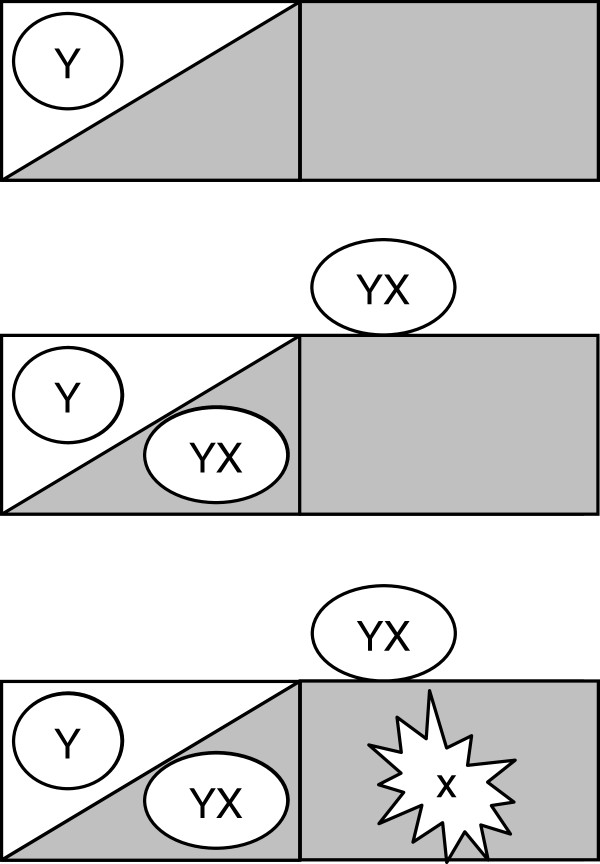
**Two hypothetical classes of coexisting proteins, X and Y.** Proteins of class X are essential for ecological adaptations, and proteins of class Y are essential for basic biological processes. This schematic picture shows the evolutionary importance of the proteins of each class for ecological adaptation and survival. Organisms having proteins Y can live only in the water environment (upper fig.). Organisms possessing proteins Y and X can exist in the water, in the terrestrial environment and in the mud (central fig.). An organism that loses its Y proteins in the terrestrial environment is evolutionarily eliminated (bottom fig.).

To address variations in functional significance, a transition between the following two coexisting classes was postulated for the proteins: the class of optional proteins that are essential for ecological adaptations, which naturally emerge and are eliminated during evolution, and the class of proteins essential for basic life processes, which are protected from immediate loss (Figure 1). Proteins involved in photosynthesis are good examples of proteins from the first class. These proteins are essential for the life of plants. In contrast, the proteins are not essential for the life of parasitic organisms having plant origins. For example, apicomplexan parasites (such as the malaria parasites Plasmodium) carry a plastid-like genome with greatly reduced sequence complexity and have an obvious plant origin. Such parasites are certainly not able to perform photosynthesis [19,20]. Examples of the proteins from the second class are the proteins involved in the process of transcription.

Two sources for new interactions were considered: one, newly emerging proteins and two, proteins within the currently existing interactome. The overall preference for gaining new interactions was assumed to be related to the node degree. In addition, two methods for losing new interactions were considered; the first was related to protein deactivation, and the second one was spontaneous. Because there is evidence that more important proteins evolve similarly to others [21], the kinetic parameters for the evolution of the number of interaction partners were assumed to be independent of protein class.

The described model predicts a double-shifted power-law distribution for the node degree. Therefore, it confirms the earlier proposal of a double exponential distribution for the node degree [22] in the range of small degrees. The model also reveals parabolic relationships between the total number of interactions and the total number of interacting proteins. The parameters of the derived mathematical formulas were estimated by fitting the theoretical predictions of the model to the existing data for the interactomes of 10 different species. This model enabled us to reveal the evolutionary kinetics of proteome formation, the differentiation process and the process of gaining new interactions.

## Results

### Kinetic model of the evolution of a protein interaction network

#### Proteome formation

**Figure 2:**
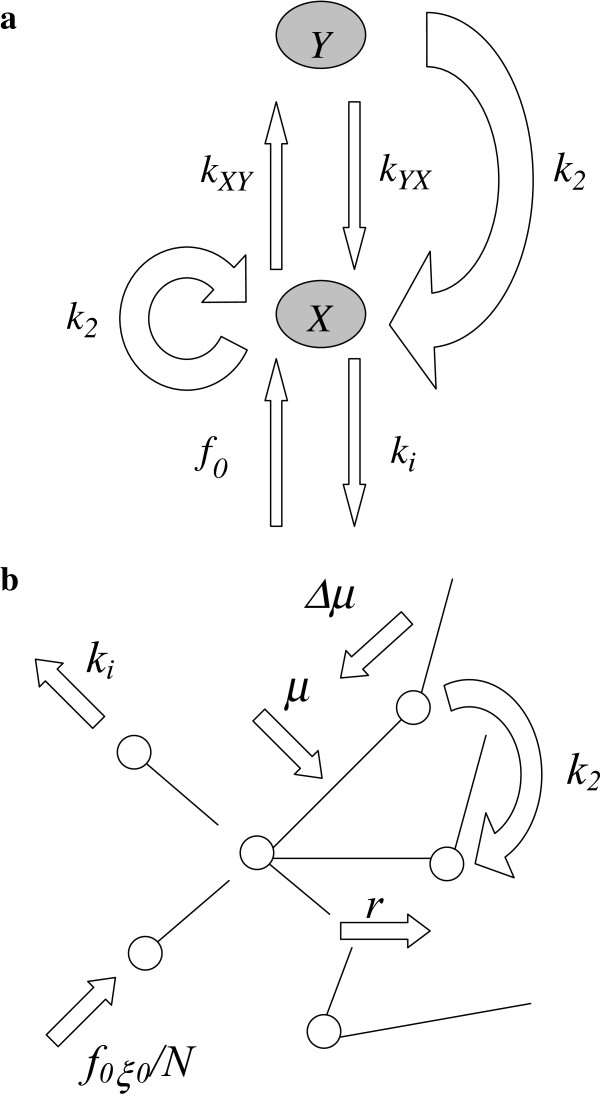
**The kinetic model of the protein interactome evolution. a**. Schematic representation of proteome formation. **b**. Schematic representation of interactome formation. The symbols *X* and *Y* indicate the protein class. Other symbols indicate rates: *f*_*0*_ - protein origination, *k*_*i*_ - protein inactivation, *k*_*XY*_ and *k*_*YX*_ - protein transition between classes, *k*_*2*_ - protein duplication, *f*_0_*ξ*_0_/*N* - emergence of an interaction with a newborn protein, *μ* emergence of an interaction with an existing protein, Δ*μ*- preference and *r –* loss of interaction.

Let us consider two classes of proteins, X and Y, which are evolving according to the following rules (for details, see the Methods). New proteins of class X originate at rate *f*_*0*_ and are inactivated at rate *k*_*i*_ (Figure 2a). These proteins are transferred to class Y at rate *k*_*XY*_. Proteins of class Y are transferred back to class X at rate *k*_*YX*_*.* These proteins, which are essential for cell function, are not inactivated directly. All proteins are duplicated at rate *k*_*2*_, but the duplicates of the Y class belong to class X. These rules are included in the set of eqs. 1 and 2, describing the variation in the size of population *X* and *Y* (continuous approach), with time *t*:

(1) $$\frac{d}{\text{dt}}X=f_{0}-k_{i}X-k_{\text{XY}}X+k_{\text{YX}}Y+k_{2}\left(X+Y\right)$$

(2) $$\frac{d}{\text{dt}}Y=k_{\text{XY}}X-k_{\text{YX}}Y$$

All parameters of the model describing the rates are treated as fixed.

#### Interactome formation

By definition, the node degree *ξ* is the number of node interaction partners. Let us assume that a single protein gains new interactions with newly emerging proteins at the rate *f*_0_*ξ*_0_/*N*, where *ξ*_*0*_ is the degree of an entirely new protein and *N* is the total number of interacting proteins (Figure 2b). This protein also gains new interactions within the existing interactome - at the rate *μ*. The process of gaining new interactions is preferential in a manner that enhances the rate of gain by Δ***ε*** per unit increase in *ξ*. The interactions are duplicated with the duplication of interacting partners at the rate *k*_*2*_ and are also lost with partner inactivation at the rate *k*_*i*_(1 − *Y*/*N*)*ξ* or spontaneously at the rate *r*. The above can be described quantitatively by eq. 3

(3) $$\begin{array}{l} \frac{\mathrm{d\xi}}{\mathrm{d\tau}}=f_{0}\xi_{o}/N+\mu \left(N-1-\xi \right)+\Delta \varepsilon \xi +k_{2}\xi \\ \;-k_{i}\left(1-Y/N\right)\xi -\mathrm{r\xi} \end{array}$$

where τ is the protein age.

For simplicity, only the proteins that emerged in the steady state of proteome formation (*dX/dt = 0*, *dY/dt = 0*) were analyzed in the following.

Then, the resolution of eq. 3 describing the evolution of a protein’s node degree is

(4) $$\xi =\left(\xi_{r}+\xi_{0}\right)\text{Exp}\left[\mathrm{v\tau}\right]-\xi_{r}$$

where *ξ*_*r*_ is presented as it is defined in the Methods.

#### Node degree distribution

As mentioned above, the degree of a node (protein) in a network (interactome) is the number of links (interactions) to other nodes, or simply the number of contacts. Its statistical variety may be described by the node degree distribution, i.e., the mathematical function indicating the number of nodes with a given degree. In a continuous approach, the discussed function is denoted as *dn/dξ* and can be obtained by considering the small number of synchronized proteins evolving with age and the age-dependent node degree. As shown in the Methods, this leads to

(5) $$\frac{\text{dn}}{\mathrm{d\xi}}=A_{1}{\left(1+\xi /\xi_{r}\right)}^{-\beta_{1}}+A_{2}{\left(1+\xi /\xi_{r}\right)}^{-\beta_{2}}$$

The amplitudes *A*_*i*_ and the powers *β*_*i*_ (i = 1,2) are defined in the Methods.

#### Total number of links

Integrating *ξ* weighted by the distribution *dn/dξ* and divided by 2 gives the relation between the total number of links *L* and the total number of interacting proteins in the steady state *N*_*∞*_.

(6) $$L=p_{1}N_{\infty }+p_{2}\left(N_{\infty }-1\right)N_{\infty }/2$$

The probabilities *p*_*i*_ (i = 1,2) are defined in the Methods.

The cited quantities *ξ*_*r*_, *A*_*i*_, *β*_*i*_, *p*_*i*_ can be related to reality by fitting eqs. 5 and 6 to experimental data. However, they are dependent on the kinetic parameters of the processes considered in the model (see the Methods). This approach may lead to the quantitative estimation of these parameters.

### Computer simulations

#### Experimental data

The values of *N*_∞_ and *L* and the references for the considered experimental interactomes are summarized in Table 1. Only single protein-protein interaction records (without self-interactions) were analyzed. No non-interacting proteins were reported.

**Table 1:** Experimental data for the studied interactomes

** Interactome**	***N***_***∞***_	***L***	**Database**
*A. thaliana*	487	959	BIND
*B. taurus*	129	107	DIP
*C. elegans*	3227	5026	BIND
*D. melanogaster*	7910	23128	BIND
*E. coli*	399	312	BIND
*H. pylori*	724	1403	COSIN
*H. sapiens*	2529	3376	DIP
*M. musculus*	1003	994	DIP
*R. norvegicus*	349	304	DIP
*S. cerevisiae*	4135	7839	COSIN

#### Fitting the model of the node degree distribution to the experimental data

**Figure 3:**
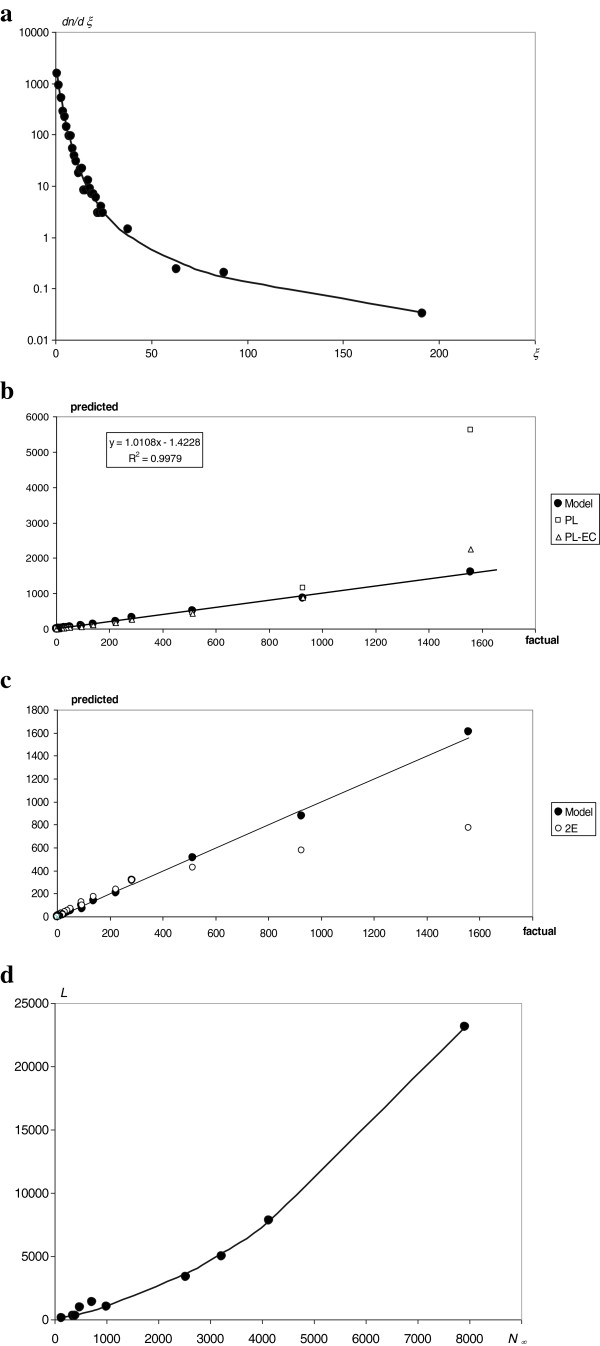
**Fitting of the kinetic model to the experimental data. a**. The result of fitting the model of the node degree distribution to the *log* of the experimental node degree histogram for the *S. cerevisiae* protein interaction network. Axis *ξ* - the node degree; axis *dn/dξ* - the number of proteins of a given node degree. The dots represent the database data. The last four points indicate the average value of the node degree and the centers of an arbitrarily defined range. The continuous line connects the theoretical predictions of the model. **b**. Comparison of the fit using the proposed model (eq. 5) and the fits using other models: PL - power-law model and PL-EC – generalized power-law with exponential cut-off model. The continuous line indicates a linear trend for the factual values and the values predicted by our model. The parameters of the trend line are shown in the inset. **c**. Comparison of the fit using the current model (eq. 5) and the fits using our previous double exponential model, 2E [20]. The continuous line indicates the ideal fit (y = x). **d**. The result of fitting the model of the dependence of *N*_*∞*_ and *L* to the points representing values for 10 different interactomes*.* The dots represent the database data. The continuous line connects theoretical predictions of the model.

The *Mathematica 4.1* standard procedure NonlinearRegress, from the package Statistics`NonlinearFit`, was applied to fit the proposed model (eq. 5) to the experimental distribution obtained by the statistical analysis of the records for the *S. cerevisiae* interactome (Table 1)*.* The results of the fitting, i.e., the values of the quantities *A*_*i*_, *β*_*i*_ and *ξ*_*r*_, are presented in Table 2. The corresponding experimental points and fitted distribution are presented in Figure 3a. The mean relative error of fit to the data points equals 0.17 and is smaller than that of other comparative fits that were performed, namely the power law (PL), *~ξ*^*-c*^ (0.34), and the generalized power law with exponential cut-off (PL-EC), *~*(*ξ + c*_*1*_)^*-c2*^ e^-*ξ/c3*^ (0.24). A detailed comparison of the different fits is shown in Figure 3b. The correlation coefficient for the fitting performed with our model (eq. 5) is 0.999. For the PL model and PL-EC models, it is 0.918 and 0.979, respectively. A comparison of the fits using the current model and our previous double exponential model is presented in Figure 3c.

**Table 2:** The results of fitting the model to the experimental data

**Quantity**	** Estimate**	**SE (%)**
*A*_1_	3184.82	32
*A*_2_	49.8628	77
*β*_1_	4.80485	29
*β*_2_	2.1242	20
*ξ*_*r*_	6.30779	51
*p*_1_	0.73526	13
*p*_2_	0.000552383	5

#### Fitting the model of the dependence of N_∞_ and L to the experimental data

The *Mathematica 4.1* standard procedure (NonlinearRegress), from the package Statistics`NonlinearFit`, was applied to fit the proposed model (eq. 6) to the set of (*N*_*∞*_, *L*) pairs for 10 different interactomes (Table 1). The results of the fitting, i.e., the values of the quantities *p*_*i*_, are listed in Table 2. The corresponding experimental points and fitted plot are presented in Figure 3d.

#### Finding the values of the kinetic parameters of the model

The general parameters of both the node degree distribution (*A*_*i*_, *β*_*i*_ and *ξ*_*r*_) and the total number of links (*p*_*i*_) can be related to the parameters of the kinetic model. Using both sets of parameters increases the universality and the credibility of the final estimated parameters of model.

A random-walking-type algorithm was developed to estimate the values of the kinetic parameters *k*_*i,*_*k*_*2,*_*k*_*XY,*_*k*_*YX,*_*ξ*_*0,*_*μ*_*,*_*Δε* and *r,* thus determining the values of the quantities *A*_*i*_, *β*_*i*_, *ξ*_*r*_ and *p*_*i*_ (see Methods). The results of both former simulations were joined, and the error measure *χ*^*2*^, defined below, was minimized:

(7) $$\begin{array}{l} \chi^{2}=\frac{{\left(\xi \;{"}_{r}-\xi {'}_{r}\right)}^{2}}{{\left(\mathrm{SE\xi}{'}_{r}\right)}^{2}}\\ \;+\sum_{i=1}^{2}\left(\frac{{\left(A\;{"}_{i}-A{'}_{i}\right)}^{2}}{{\left(\mathrm{SEA}{'}_{i}\right)}^{2}}+\frac{{\left(\beta \;{"}_{i}-\beta {'}_{i}\right)}^{2}}{{\left(\mathrm{SE\beta}{'}_{i}\right)}^{2}}+\frac{{\left(p\;{"}_{i}-p{'}_{i}\right)}^{2}}{{\left(\mathrm{SEp}{'}_{i}\right)}^{2}}\right) \end{array}$$

In the above equation, the singly primed values (‘) were taken from Table 2, and the doubly primed values (“) were calculated according to the formulas in the Methods, which contain kinetics parameters. Finally, the calculated values of the kinetic parameters led to the estimation of the parameter *f*_0_, which was used in the equation:

(8) $$f_{0}=N_{\infty }\left(\left(1-\kappa \right)k_{i}-k_{2}\right)$$

with the assumption that *N*_∞_ = 4135, as for *S. cerevisiae*.

At minimization, a few additional simple constraints were added to eliminate the kinetic parameters that showed no real physical importance. Several attempts were made, and the results of the best minimization courses are presented in Table 3.

**Table 3:** Estimated kinetic parameters of the model

** Kinetic** **parameter**	**Best estimation** *χ*^2^ = 0.14	** Average** (the 10 best)	** SE (%)** (the 10 best)
*k*_*i*_	8.61692	12.683567	10.1
*k*_*2*_	0.122669	0.052556819	27.7
*k*_*XY*_	0.0611168	0.09274057	10.4
*k*_*YX*_	2.8542	4.249004	10.1
*ξ*_*0*_	0.426372	0.4256051	0.1
*μ*	0.00237779	0.003526257	10.1
*Δ****ε***	10.6696	15.91212	9.9
*r*	0.107993	0.204924912	45.9
*f*_*0*_	34376.8	51108.95	10.1

#### Simulations of the kinetics of the protein interactome evolution

**Figure 4:**
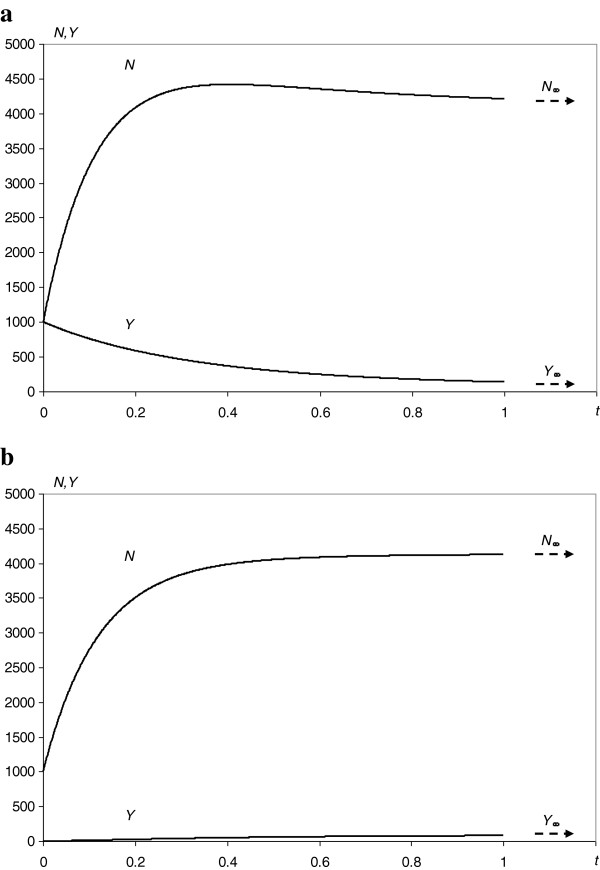
**Simulations of the evolution of the proteome. a**. It was also assumed that at the beginning of the evolution, all proteins were essential for life processes (*N = Y*). **b**. It was also assumed that at the beginning of the evolution, there were no proteins essential for life processes (Y = 0). Axis *t* indicates time, and *N* and *Y* indicate the total number of proteins and the number of proteins essential for life processes, respectively. The continuous line represents the theoretical predictions of the model.

**Figure 5:**
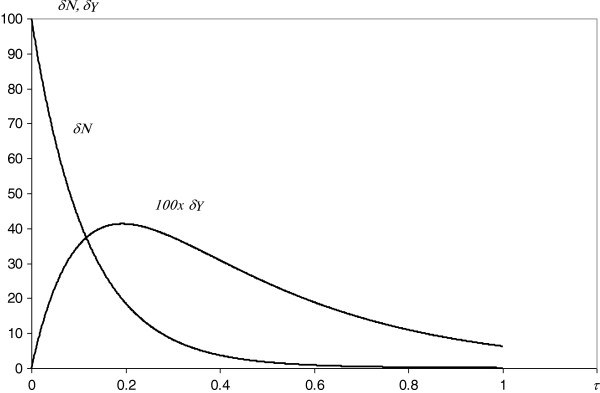
**Simulation of the evolution of a small sample of synchronized proteins.** The hypothetical kinetics of the proteome evolution are shown. Axis *τ* represents the protein age, and δ*N* and δ*Y* indicate the small number of proteins (initial number 100) and the fraction of essential ones (multiplied by 100 for better resolution), respectively. Continuous line – theoretical predictions of the model.

**Figure 6:**
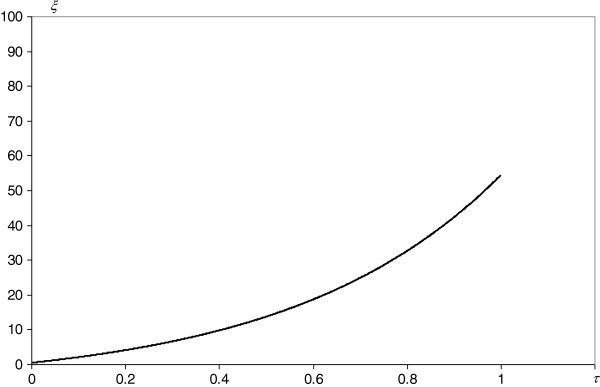
**Simulation of the evolution of a single node (protein) degree.** Axis *τ* - node age, axis *ξ* - node degree.

Using eqs. A.1 and A.2, A.6 and A.7 and eq. A.27 with the best fit parameters from Table 3, the following evolutions were simulated: the proteome (Figure 4a,b), a small sample of synchronized proteins (Figure 5) and a single protein node degree (Figure 6).

### Summary of the most important results

The proposed kinetic model (Figure 2a,b) of the evolution of a protein interaction network agrees very well with the experimental data. The node degree distribution of *S. cerevisiae* (Figure 3a) and the nonlinear dependence of the total number of links on the total number of interacting proteins (Figure 3d) can be successfully described with the derived theoretical formulas (eqs. 5 and 6). Thus, amplitudes, powers and probabilities (Table 2) were obtained according to the model in the Methods. In addition to providing a non-trivial explanation of the recently observed picture of the node degree distribution or the *N*_*∞*_ and *L* dependence, these values led to the estimation of the kinetic parameters (Table 3) of the dynamic processes governing the evolution, differentiation and cross-linking of the protein interaction network. Finding these parameters enables numerical simulations of the evolution of the following: the total proteome (Figure 4a,b), the decrease and differentiation of a small sample of synchronized proteins (Figure 5) and the expansion of a single protein node degree (Figure 6). The estimated characteristic times of evolution are 1/*γ*_1_ = 0.12, 1/*γ*_2_ = 0.35 and 1/*v* = 0.45, indicating that the evolution of the node degree is slower than the evolution of the proteome. The estimated fraction of essential proteins *κ* = *Y*_*∞*_/*N*_*∞*_ equals 0.02.

## Discussion

The presented kinetic model of the evolution of a protein interactome is an extension of the previous two-class model [22] describing a double exponential distribution of the node degree. The current version of the model additionally postulates asymmetry in the functional importance of the considered protein classes and takes into account a possible evolutionary transition between the classes. This model also considers gene doubling and preferential attachment.

From a cognitive point of view, the proposed model led to a satisfactory fit to the node degree histogram (Figure 3a) and to the picture of the nonlinear dependence of *N*_∞_ and *L* (Figure 3d). Moreover, the node degree fit according to the derived eq. 5 is 50% better than that of the power law [1] or 25% better than that of the generalized power law with exponential cut-off [23]. This fit is also much better than the fit from our previous double exponential model (Figure 3c), neglecting gene doubling, preferential attachment and inter-class transitions. Moreover, the current model led to the estimation of unknown values of kinetic parameters (Tables 2 and 3). Thus, this model reveals the kinetics of evolution of the interactome (Figure 4a,b), the final result of which (approaching the steady state) does not depend on the initial state of the protein’s importance. Although the evolution of the total proteome stabilizes, individual proteins are eliminated (Figure 5). Gaining new interactions from a single protein (Figure 6) is much slower than the evolution of the proteome, but the increase of protein degree with protein age confirms the trend observed for proteins of eukaryotic and post-eukaryotic origin [7].

The model and its estimated kinetic parameters allow a sketch of a hypothetical picture of proteome evolution, indicating that class Y of proteins that are functionally essential for basic processes of *S. cerevisiae* finally includes approximately 2% of protein population. One could expect that all the genes from this class and a portion of the genes belonging to the first class (proteins important for ecological adaptations) are strictly essential (their deletion is lethal). We compared this expectation with experimental results. Deutschbauer [24] and co-workers showed that the deletion of 19% of genes causes lethality. This finding is in agreement with our results. The second expectation is that some proteins belonging to the first class (proteins important for ecological adaptations) have a function only in particular conditions. This hypothesis was shown experimentally by Hillenmeyer [25] and co-workers, who performed 1144 chemical genomic assays on the yeast whole-genome heterozygous and homozygous deletion collections and quantified the growth fitness of each deletion strain in the presence of chemical or environmental stress conditions. In their first experiment, only approximately 40% of the gene deletion strains performed phenotype. However, 97% of the gene deletions also exhibited a measurable growth phenotype in one of the tested conditions. In conclusion, our results fit well to the experimental data.

In this picture, the origination of new species may be related to variations in the value of the parameters governing the kinetics of evolution (e.g., *f*_*0*_, which directly determines the value of *N*_∞_), resulting in the origination of a new steady state of proteome organization. In addition, the results indicate that entering the important class Y is approximately 50-fold slower than leaving it. This finding illustrates how difficult it is to become a member of a protein “gentlemen's club” and how easy it is to lose this position. Mechanisms of selection and adaptation certainly play an important role in this type of arrangement, ensuring stability in the composition of backbone biochemical reactions. The stability is one of the most important factors supporting organisms’ survival. During evolution, organisms investigate optimal paths of growth and replication, which is possible if and only if the organisms preserve certain optimal and stable biochemical machinery [26].

The obtained results also show how large dynamic changes involving new protein emergence and inactivation may occur in class X proteins without disturbing the steady state of the entire system. The results also revealed an essential preference for gaining new interactions. Within the interactome of *S. cerevisiae,* the first interaction of a given protein increases its rate of gaining a new one by approximately 100%.

To relate these findings to the timescale of real evolution, it is reasonable to arbitrarily assume that a unit of time in the model corresponds to 10^9^ years. Then, an *f*_*0*_ of 34376.8 means approximately 30 new proteins per 10^6^ years. Consequently, the characteristic times of proteome evolution can be estimated to equal 1.2·10^8^ and 3.5·10^8^ years. The shorter time describes the timescale of entering the “higher” class, and the longer time describes the timescale of protein deactivation. The characteristic time of gaining a new interaction is 4.5·10^8^ years.

From the perspective of describing the current distribution of protein degree or the dependence of the total number of links on the size of the interactome, a steady-state approximation for proteome evolution appears to be a correct simplification. Most of the observed proteins most likely originated during the “steady state era”. For a more precise description of the connectivity of older proteins, e.g., those from the pre-eukaryotic radiation era, the model should also take into account the variations with time in both the proteome size and the values of kinetic parameters.

One of the main predictions of the proposed model (Figure 6) is consistent with the finding that, on average, evolutionarily older proteins have more interactions with other proteins than do their younger counterparts [27]. Because the discussed model only addresses the overall PPI network evolution, the more detailed features of this process, i.e., the fast asymmetric functional divergence of duplicated genes [28] or the modular preferential attachment [18] were disregarded, offering a large simplification with no loss of prediction ability. Nevertheless, some asymmetric divergence and modularity is still contained in our model, mainly from the assumption of two different classes of protein importance.

Finally, the proposed model relates the static observables, such as the node degree distribution, to many dynamic evolutionary processes. The discussed dynamics are not a trivial consequence of the birth and death of proteins. The dynamics also involve the transition of proteins between classes, which leads to a dynamic balance, in which a given protein may change its importance class several times depending on the environmental conditions. Thus, the amplitudes in the derived formula for node degree distribution describe an effective dynamic content of each protein class but not the number of specific proteins.

As previously shown, the presented kinetic model of the evolution of a protein interaction network offers a solid foundation for future development and provides a productive research approach to protein interaction networks.

In future studies, it would be nice to have a more definitive evaluation of how the model’s simplifications affect its accuracy. Standard errors of the estimation (Table 3) show that the spontaneous loss of interactions, *r,* is statistically insignificant and is, thus, not likely to be critical for the stability of the model. Furthermore, the duplication rate, *k*_*2*_, is of less statistical significance. Possibly, these parameters could be omitted in simplifications that neglect parameters of the second order without considerable loss in the accuracy of the model.

Despite good fits, we are aware of the fact that the cited experimental methods have enormous potential for false data. The PPI data are full of false positives and false negatives, which, when unquestioningly included, tend to generate false conclusions. Necessarily, the model was applied to the data that exist. High-throughput data tend to be worse than low-throughput data [29]. We expect that the errors in the set of interactions can mainly disturb the estimation of the general parameters of the extensive type (amplitudes *A*_*i*_, probabilities *p*_*i*_). Test simulations that were performed indicate that a 10% increase in the value of those parameters may result in a change of the final estimated kinetic parameters of the model reaching up to 70%. Thus, the results may change in the face of future data.

The presented and applied model of the evolution of the protein interactome by its nature contains some abstraction, which does not invalidate the results (see Hamilton [30]). For example, the central concept of “essentiality” is a significant binary simplification of a gene's ability to survive and reproduce. In the future, this concept may be replaced by the more detailed continuous approach with the full spectrum of gene fitness. A similar school of thinking was shown in our previous paper [22], which presented multi-exponential fitting that described the full spectrum of contributions from different classes of proteins. This method also indicated the domination of the two basic subpopulations.

## Conclusions

The current model leads to a number of predictions that we can hope to test in the not-so-distant future. The most interesting findings are the following:

– A small sample of synchronized proteins decreases and differentiates; the degree of a single protein node expands.

– The evolution of a node degree is slower than the evolution of the proteome.

– The evolution of the total proteome stabilizes.

– Entering the class of proteins that are essential for basic biological processes is approximately 50-fold slower than leaving it.

– Large dynamic changes, involving new protein emergence and inactivation in class X, do not disturb the steady state of the entire system.

– There is a parabolic relationship between the total number of interactions and the total number of interacting proteins.

– The connectivity of the oldest part of the interaction network is dense; the node degree distribution follows the sum of the two shifted power-law functions.

We hope that the above paper presents a helpful advance in this interesting area.

## Methods

### Mathematical formulation of a kinetic model of the evolution of a protein interaction network

#### Proteome formation

The set of eqs. 1 and 2 (see main text) describing the rate of variation in the size of protein classes X and Y can be rewritten using a more convenient pair of variables, i.e., the total number of evolving proteins, *N* = *X* + *Y*, and the number of essential proteins, *Y*. One can obtain

(A.1) $$\frac{d}{\text{dt}}N=f_{0}-\left(k_{i}-k_{2}\right)N+k_{i}Y$$

(A.2) $$\frac{d}{\text{dt}}Y=k_{\text{XY}}N-\left(k_{\text{XY}}+k_{\text{YX}}\right)Y$$

where *f*_*0*_ is the rate of origination of entirely new proteins of class X, *k*_*i*_ is the rate of protein inactivation, *k*_*XY*_ and *k*_*YX*_ are the rates of protein migration between classes X and Y, *k*_*2*_ is protein duplication rate and *t* is the time.

The steady-state (*dN/dt = 0*, *dY/dt = 0*) values of the total number of proteins, *N*_∞_, and the number of essential proteins, *Y*_∞_, can be estimated according to eqs. 1 and A.2 as

(A.3) $$N_{\infty }=f_{0}/\left(\left(1-\kappa \right)k_{i}-k_{2}\right)$$

(A.4) $$Y_{\infty }=\kappa N_{\infty }$$

where

(A.5) $$\kappa =k_{\text{XY}}/\left(k_{\text{XY}}+k_{\text{YX}}\right)$$

Consequently, the evolution of a small sample of proteins originating within the short time period *δt* can be described by the set

(A.6) $$\frac{d}{\text{dt}}\mathrm{\delta N}=-\left(k_{i}-k_{2}\right)\mathrm{\delta N}+k_{i}\mathrm{\delta Y}$$

(A.7) $$\frac{d}{\text{dt}}\mathrm{\delta Y}=k_{\text{XY}}\mathrm{\delta N}-\left(k_{\text{XY}}+k_{\text{YX}}\right)\mathrm{\delta Y}$$

with the initial conditions

(A.8) $$\mathrm{\delta N}\left[t_{0}\right]=f_{0}\mathrm{\delta t}$$

(A.9) $$\mathrm{\delta Y}\left[t_{0}\right]=0$$

The eigenvalues, *λ*_*1*_ and *λ*_*2*_, obtained from the determinant requirement

(A.10) $$\det\left[\begin{array}{cc} -\left(k_{i}-k_{2}\right)-\lambda & k_{i}\\ k_{\text{XY}} & -\left(k_{\text{XY}}+k_{\text{YX}}\right)-\lambda \end{array}\right]=0$$

describe the characteristic rates of change in sample size

(A.11) $$\lambda_{1,2}=0.5\left(-b\pm \sqrt{b^{2}-4c}\right)$$

where

(A.12) $$b=k_{i}-k_{2}+k_{\text{XY}}+k_{\text{YX}}$$

(A.13) $$c=\left(k_{i}-k_{2}\right)\left(k_{\text{XY}}+k_{\text{YX}}\right)-k_{\text{XY}}k_{i}$$

When *b* > 0 and *b*^*2*^/4 > *c* > 0, both values of *λ* are negative, and the protein sample vanishes. Then, the decay of its total size can be described by the formula:

(A.14) $$\mathrm{\delta N}\left[t\right]=\left(a_{1}\text{Exp}\left[-\gamma_{1}\left(t-t_{0}\right)\right]+a_{2}\text{Exp}\left[-\gamma_{2}\left(t-t_{0}\right)\right]\right)f_{O}\mathrm{\delta t}$$

Where

(A.15) $$a_{1}=1/\left(1-s\right)$$

(A.16) $$a_{2}=-s/\left(1-s\right)$$

(A.17) $$s=\left(k_{i}-k_{2}+\lambda_{1}\right)/\left(k_{i}-k_{2}+\lambda_{2}\right)$$

(A.18) $$\gamma_{i}=-\lambda_{i}\;\left(i=1,\;2\right)$$

The right side of eq. A.14 describes the number of proteins aged between *τ = t-t*_*0*_ and *τ = t-t*_*0*_ *+ δt* that are observed at the moment *t.* Thus*,* the density of the distribution of protein age, *dn/dτ* can be described by

(A.19) $$\frac{\text{dn}}{\mathrm{d\tau}}=\left(a_{1}\text{Exp}\left[-\gamma_{1}\tau \right]+a_{2}\text{Exp}\left[-\gamma_{2}\tau \right]\right)f_{0}$$

#### Interactome formation

Considering the protein degree *ξ*, i.e., the number of partners with which the protein interacts within the interactome network, and according to eq. 3 (see main text), for a protein emerging in the steady state regime (*dN/dt = 0*, *dY/dt = 0*), we can write

(A.20) $$\frac{\mathrm{d\xi}}{\mathrm{d\tau}}=g+\mathrm{v\xi}$$

where

(A.21) $$g=f_{0}\xi_{0}/N_{\infty }+\mu \left(N_{\infty }-1\right)$$

(A.22) $$v=\Delta \varepsilon +k_{2}-k_{i}\left(1-\kappa \right)-r-\mu$$

where *ξ*_*0*_ is the degree of an entirely new protein, *μ* is the rate of an emerging new interaction within the proteome, Δ***ε*** is the increase in the rate per link resulting from the preference effect, *r* is the rate of interaction loss and *μ* is the protein age. The meaning of the other symbols is the same as that previously stated. *N*_∞_ and *κ* are described by equations A.3 and A.5.

Then,

(A.23) $$\xi =\left(\xi_{r}+\xi_{0}\right)\text{Exp}\left[\mathrm{v\tau}\right]-\xi_{r}$$

where

(A.24) $$\xi_{r}=g/v$$

Combined with A.23, it is easy to show that

(A.25) $$\tau =\mathrm{Ln}\left[w\left(1+\xi /\xi_{r}\right)\right]/v$$

where

(A.26) $$w=\xi_{r}/\left(\xi_{r}+\xi_{0}\right)$$

#### Node degree distribution

The degree distribution of a protein node, *dn/dξ*, can be obtained by transformation of the derivative A.19 replacing the variables *τ* and *ξ*, described by eq. A.25. According to the formula

(A.27) $$\frac{\text{dn}\left[\xi \right]}{\mathrm{d\xi}}=\frac{\text{dn}\left[\tau \right]}{\mathrm{d\tau}}\left|{{}_{\tau =\tau }}_{\left[\xi \right]}\right)\frac{\mathrm{d\tau}\left[\xi \right]}{\mathrm{d\xi}}$$

one can obtain

(A.28) $$\frac{\text{dn}}{\mathrm{d\xi}}=A_{1}{\left(1+\xi /\xi_{r}\right)}^{-\beta_{1}}+A_{2}{\left(1+{\xi /\xi }_{r}\right)}^{-\beta_{2}}$$

where

(A.29) $$A_{i}=a_{i}w^{-\beta_{i}+1}f_{O}/\left(\xi_{r}v\right)\;\left(i=1,2\right)$$

(A.30) $$\beta_{i}=\gamma_{i}/v+1\;\left(i=1,2\right)$$

For *ξ*/*ξ*_*r*_ < < 1, the distribution A.28 can be approximated by a double exponential formula

(A.31) $$\frac{\text{dn}}{\mathrm{d\xi}}=A_{1}\text{Exp}\left[-\varepsilon_{1}\xi \right]+A_{2}\text{Exp}\left[-\varepsilon_{2}\xi \right]$$

where

(A.32) $$\varepsilon_{i}=\beta_{i}/\xi_{r}\;\left(i=1,2\right)$$

#### Total number of links

For a protein emerging in the steady state (*dN/dt = 0*, *dY/dt = 0*), the total number of links can be estimated by the formula

(A.33) $$L=0.5\int_{\xi_{0}}^{\infty }\frac{\text{dn}}{\mathrm{d\xi}}\mathrm{\xi d\xi}$$

Using distribution A.28 and equations A.3, A.21, A.24, A.26 and A.29 one can obtain

(A.34) $$L=p_{1}N_{\infty }+p_{2}\left(N_{\infty }-1\right)N_{\infty }/2$$

where

(A.35) $$p_{1}=\frac{\phi }{2\nu }\left(\frac{a_{1}\left(\frac{\gamma_{1}}{v}+\frac{\phi }{v}\right)}{\left(\beta_{1}-1\right)\left(\beta_{1}-2\right)}+\frac{a_{2}\left(\frac{\gamma_{2}}{v}+\frac{\phi }{v}\right)}{\left(\beta_{2}-1\right)\left(\beta_{2}-2\right)}\right)\xi_{0}$$

(A.36) $$p_{2}=\frac{\phi }{\nu }\left(\frac{a_{1}}{\left(\beta_{1}-1\right)\left(\beta_{1}-2\right)}+\frac{a_{2}}{\left(\beta_{2}-1\right)\left(\beta_{2}-2\right)}\right)\frac{\mu }{v}$$

and

(A.37) $$\phi =\left(1-\kappa \right)k_{i}-k_{2}$$

## Competing interests

The authors declare that they have no competing interests.

## Authors’ contributions

PHP proposed the model and performed model-based analysis. PHP, SK, and PZ participated in the design of the study. PP and SK drafted the manuscript. PP and PZ revised the manuscript. All authors read and approved the final manuscript.
